# Harnessing sequencing data for porcine reproductive and respiratory syndrome virus (PRRSV): tracking genetic evolution dynamics and emerging sequences in US swine industry

**DOI:** 10.3389/fvets.2025.1571020

**Published:** 2025-03-06

**Authors:** Srijita Chandra, Guilherme Cezar, Kinath Rupasinghe, Edison Magalhães, Gustavo S. Silva, Marcelo Almeida, Bret Crim, Eric Burrough, Phillip Gauger, Darin Madson, Joseph Thomas, Michael Zeller, Jianqiang Zhang, Rodger Main, Albert Rovira, Mary Thurn, Paulo Lages, Cesar Corzo, Matthew Sturos, Kimberly VanderWaal, Hemant Naikare, Franco Matias-Ferreyra, Rob McGaughey, Jamie Retallick, Sara McReynolds, Jordan Gebhardt, Angela Pillatzki, Jon Greseth, Darren Kersey, Travis Clement, Jane Christopher-Hennings, Beth Thompson, Jonah Perkins, Melanie Prarat, Dennis Summers, Craig Bowen, Joseph Boyle, Kenitra Hendrix, James Lyons, Kelli Werling, Andreia G. Arruda, Mark Schwartz, Paul Yeske, Deborah Murray, Brigitte Mason, Peter Schneider, Samuel Copeland, Luc Dufresne, Daniel Boykin, Corrine Fruge, William Hollis, Rebecca Robbins, Thomas Petznick, Kurt Kuecker, Lauren Glowzenski, Megan Niederwerder, Xiaoqiu Huang, Daniel C. L. Linhares, Giovani Trevisan

**Affiliations:** ^1^Department of Computer Science, Iowa State University, Ames, IA, United States; ^2^Veterinary Diagnostic and Production Animal Medicine, Iowa State University, Ames, IA, United States; ^3^Veterinary Population Medicine, University of Minnesota, Saint Paul, MN, United States; ^4^Kansas Veterinary Diagnostic Laboratory, Kansas State University, Manhattan, KS, United States; ^5^Kansas Department of Agriculture, Manhattan, KS, United States; ^6^Department of Diagnostic Medicine/Pathobiology, Kansas State University, Manhattan, KS, United States; ^7^Veterinary & Biomedical Sciences Department, South Dakota State University, Brookings, SD, United States; ^8^South Dakota Animal Industry Board, Pierre, SD, United States; ^9^Ohio Animal Disease and Diagnostic Laboratory, Reynoldsburg, OH, United States; ^10^College of Veterinary Medicine, Purdue University, West Lafayette, IN, United States; ^11^Indiana State Board of Animal Health, Indianapolis, IN, United States; ^12^Department of Veterinary Preventive Medicine, College of Veterinary Medicine, The Ohio State University, Columbus, OH, United States; ^13^Schwartz Farms Inc., Sleepy Eye, MN, United States; ^14^Swine Vet Center, St. Peter, MN, United States; ^15^New Fashion Pork, Jackson, MN, United States; ^16^Country View Family Farms, Hatfield, PA, United States; ^17^Innovative Agriculture Solutions, LLC, Ames, IA, United States; ^18^Prestage Farms, St. Pauls, NC, United States; ^19^Swine Veterinary Partners, Quebec, CA, Canada; ^20^Smithfield Foods, Smithfield, VA, United States; ^21^The Maschhoffs LLC, Carlyle, IL, United States; ^22^Carthage Veterinary Service LTD, Carthage, IL, United States; ^23^Pig Improvement Company, Hendersonville, TN, United States; ^24^ArkCare, Omaha, NE, United States; ^25^The Hanor Company, Enid, OK, United States; ^26^Pipestone Veterinary Services, Pipestone, MN, United States; ^27^Swine Health Information Center, Ames, IA, United States

**Keywords:** PRRSV, database, BLAST, epidemiology, surveillance, genetics

## Abstract

Porcine reproductive and respiratory syndrome virus (PRRSV) is the most important swine pathogen affecting the United States of America (USA), leading to significant economic losses. Despite advances in diagnostic testing, there remains a gap in understanding the genetic evolution of PRRSV, especially in tracking the emergence of novel sequences and their spread across different regions and production stages. This research addresses this gap by developing a systematic methodology for directly collecting and analyzing PRRSV ORF5 sequences from veterinary diagnostic laboratories. The study aimed to identify trends among collected sequences and emerging PRRSV sequences by integrating nucleotide sequence data with metadata, providing critical insights into their geographic distribution, collected specimens, swine age groups, lineages, variants, and restriction fragment length polymorphism (RFLP) patterns. As of December 2024, the database housed 115,643 PRRSV ORF5 sequences. Sublineages 1B, 1A, 1H, and 1C.5 were the major wild-type PRRSV sequences detected over time, whereas vaccine-like strains comprised mostly of sublineages 5A and 8A. A novel sequence detection system was implemented, categorizing sequences based on similarity thresholds, ambiguities, and length criteria, identifying 167 novel sequences for the period between 2010 and 2024, whereas only three had continued detection in the field over time, forming clusters of detection. The analysis of these novel sequences highlighted significant trends, including the dominance of grow-finish animals in sequence origin and the high number of detections of sublineage 5A. Production sites located in states with the largest swine inventory have contributed to the most frequent detection of new PRRSV strains. Additionally, the development of a web-based tool provides end users with the capability to search sequences similar to their query sequence, providing macroepidemiological information and genetic sequence features to support PRRSV management and control. Real-time PRRSV sequencing data analysis informs producers and veterinarians of any upcoming novel sequences and trends of detection. The findings are intended to enhance current surveillance efforts and support more effective strategies for managing PRRSV outbreaks, ultimately safeguarding animal health, economic sustainability in the swine industry, and ultimately contributing to national food production sovereignty through pork-derived products.

## 1 Introduction

Porcine reproductive and respiratory syndrome virus (PRRSV) is an RNA virus affecting swine populations worldwide. The virus can be classified into two distinct species: PRRSV-1 (*Betaarterivirus europensis*), which was first reported in Europe in the 1990s, and PRRSV-2 (*Betaarterivirus americense*), which was first reported in North America in the 1980s (1–5). At present, both species can be found in several countries, and the virus is the cause of substantial economic losses in swine production systems globally. PRRS is considered the most economically important endemic swine disease in the United States (US), causing annual losses of more than $1.2 billion (6–8). According to the 2017 US Census of Agriculture, there were 66,439 pig farms in the US (9), highlighting the vast scale of swine production farms that could be potentially affected by PRRSV outbreaks.

Due to the highly infectious nature of PRRSV and associated high economic effects due to a disease named after the virus, researchers, producers, and veterinarians are highly interested in better identifying geographical distribution patterns and detecting occurrences of PRRSV to help implement disease control mechanisms as required (10). There are several projects which were developed to respond to this need. In the US, one of the earliest of such projects is the Morrison (11, 12) Swine Health Monitoring Project (MSHMP, https://mshmp.umn.edu/), which reports to project participants and producers weekly and yearly PRRS cumulative incidence (13–15) as well as site-level PRRS prevalence over time as reported by program participants (16). When comparing MSHMP trends over time, there was an increase from approximately 15% in 2009 to 30% in 2024 of breeding herds classified as positive unstable, i.e., having the presence of PRRSV and weaning positive PRRSV piglets.

Another project is the Swine Disease Reporting System (SDRS, https://www.fieldepi.org/SDRS), which aggregates data from multiple veterinary diagnostic laboratories (VDLs), and reports the information findings to stakeholders such as veterinarians, producers, and researchers (10). SDRS aims to be a source of standardized information that aggregates diagnostic data from participant VDLs, analyzes data, and reports findings in a consistent, routine, and timely fashion (10). Currently, the SDRS integrates diagnostic data and performs data analysis on an array of swine pathogens such as PRRSV-1, PRRSV-2, porcine epidemic diarrhea virus (PEDV), porcine deltacoronavirus (PDCoV), transmissible gastroenteritis (TGEV), *Mycoplasma hyopneumoniae*, porcine circovirus type 2 (PCV2), porcine circovirus type 3 (PCV3), and influenza A virus (IAV) detection by polymerase chain reaction (PCR) or reverse transcription PCR (RT-PCR) testing assays (10, 17, 18).

Diagnostic testing for PRRSV primarily relies on RT-PCR testing, which may be followed by Sanger sequencing targeting the recovery of the open reading frame-5 (ORF-5) gene (19) on selected positive samples. Sanger sequencing is a well-established method for accurately determining nucleotide gene sequences. This technique focuses on recovering the ORF5 gene of the PRRSV genome, which is used to track PRRSV viral epidemiology in North America (20). ORF5 encodes the major envelope glycoprotein 5 (GP5) and has been used widely to study the genetic diversity of PRRSV-1 and PRRSV-2 (11, 12, 21, 22), both present in the US (10). PRRSV ORF5 sequences are further classified according to restriction fragment length polymorphism (RFLP) based on enzyme cut patterns (23) and are further refined through lineage analysis, which provides an understanding of the genetic diversity and evolutionary relationships among PRRSV. Lineage classification plays an important role in PRRSV type identification due to its dependency on the molecular phylogeny of the virus (11, 12, 20, 24, 25). The lineage system was further refined into a fine-scale classification scheme for PRRSV-2 variants within the US, whereas variants are defined as genetic clusters of closely related ORF5 sequences at finer scales than sub-lineage (26). Early identification and stakeholder awareness of new emerging variants can help in the decision-making process for disease control and management to mitigate clinical disease impact and decrease economic losses. The ORF5 gene is also instrumental in distinguishing between wild-type strains and those derived from modified-live vaccines. This differentiation is crucial for monitoring the spread of wild-type strains in the field and assessing their impact on PRRSV epidemiology.

While the SDRS performs data analysis of reported RT-PCR and test results to monitor PRRSV detection (10, 27, 28), it has historically lacked the capability to capture and analyze PRRSV ORF5 genetic sequences. This limitation has left a critical gap in understanding the genetic evolution of PRRSV within the US. Without sequence data, monitoring the emergence of new variants or tracking the spread of specific lineages across diverse geographical regions and production systems is challenging. This gap leaves important questions unanswered, such as which farm types are more susceptible to the emergence of novel sequences, which states exhibit higher occurrences of novel sequences, and what lineages, variants, and RFLP patterns these novel sequences belong to. Additionally, it is crucial to determine if these sequences are a one-time event or if novel sequences show temporal variations with an increasing number of detections forming groups that could have significant field relevance. This research aimed to implement a methodology for systematically collecting and characterizing of PRRSV ORF5 sequences according to lineages, variants, and RFLP patterns. The second objective was to detect and analyze novel sequences entering the database and to identify new trends in detection among emerging PRRSV variants. A third objective was to create a web-based platform to allow users to provide a given PRRSV ORF5 sequence and search for sequences present in the database similar to the given query sequence.

## 2 Materials and methods

### 2.1 Data collection

**Figure 1:**
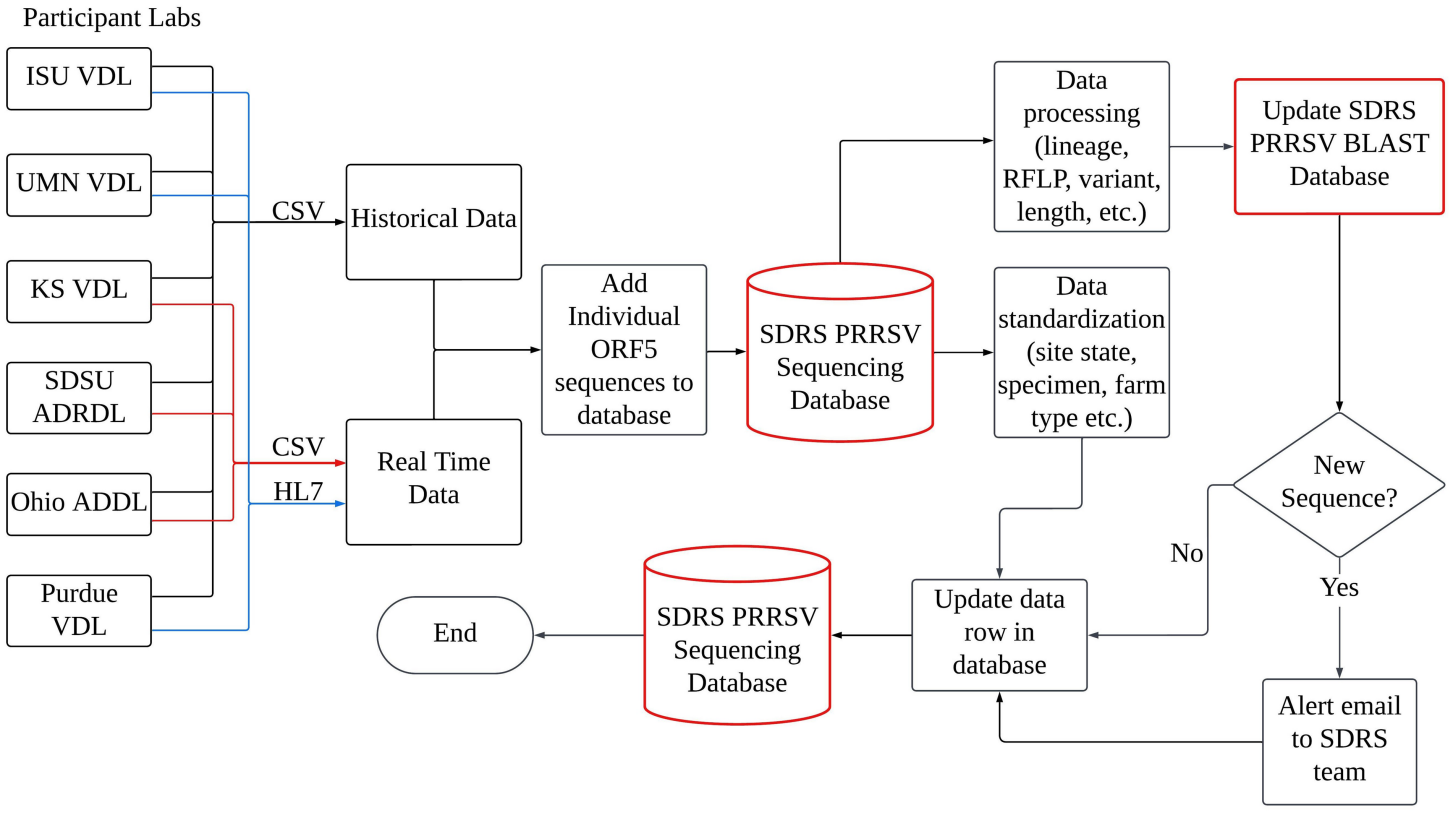
The data accumulation, processing, and updating mechanisms of the SDRS PRRSV Sequencing Database involve a sophisticated interplay between participating laboratories and the database infrastructure. Laboratories transmit their real-time sequencing data either through Comma Separated Value (CSV) files, denoted by red lines, or via Health Level Seven (HL7) messaging, represented by blue lines. This collaborative effort ensures a continuous flow of data, facilitating real-time updates and maintenance of a comprehensive repository for PRRSV genetic information.

PRRSV ORF5 sequences and associated metadata such as received date, swine age group, specimen, and site state from a consortium of six participant veterinary diagnostic laboratories (VDLs): Iowa State University (ISU), University of Minnesota (UMN), and Kansas State University (KS) respective VDLs, South Dakota State University Animal Disease Research & Diagnostic Laboratory (SDSU ADRDL), Ohio Animal Disease Diagnostic Laboratory (Ohio ADDL), and Purdue University ADDL were retrieved and included in this work. The participant laboratories perform greater than 97% of all PRRSV ORF5 sequencing done in the National Animal Health Laboratory Network (NAHLN). Sequence data had received date from January 1st, 2006, to December 31st, 2024. Given the volume and complexity of the collected data, a centralized repository was necessary and implemented to efficiently receive, manage, collate, and analyze the information. This led to developing the SDRS PRRSV sequencing database, a specialized Microsoft Structured Query Language (SQL) server database hosted on the secure, scalable server infrastructure at the ISU Veterinary Diagnostic and Production Animal Medicine Department (ISU VDPAM). The construction of the database involved two primary phases: data acquisition and data processing. The first phase focused on gathering and consolidating data from multiple sources in a retrospective and prospective fashion, while the second phase involved processing and standardizing the data to ensure uniformity for incorporation into the database. These steps were critical in preparing the data for subsequent analysis, enabling the generation of valuable insights, such as identifying predominant lineages, variants, RFLPs, and regions with high PRRSV activity (Figure 1).

A key focus during the data acquisition phase was automating the collection process to minimize human error. Two primary methods were used: Health Level Seven (HL7) messaging and Comma Separated Value (CSV) file transfers. The data was organized into historical and real-time streams. Historical data, comprising information collected prior to the establishment of the SDRS PRRSV sequencing database in 2023, was shared by participant VDLs in a CSV format (Figure 1).

Real-time data, on the other hand, was and continues to be transmitted from ISU, UMN VDL, and Purdue University using HL7 messaging, enabling continuous data sharing directly to the SDRS HL7 database. The system accessed the relevant data from ISU VDPAM through a designated data view, with SQL queries designed to extract only the necessary columns. The SQL queries were scheduled to run automatically once daily, continuously checking for new data exposed through the data view to facilitate dynamic integration.

In parallel, the CSV file transfer method involved data being transferred via email from participant VDLs to the SDRS project. KS VDL provided, and continues to provide, weekly CSV files with PRRSV ORF5 sequencing data, while SDSU ADRDL and Ohio ADDL submitted and have been submitting files monthly. The CSV files were manually moved to specific Box folders, where automated daily checks were scheduled to identify and process new files. This scheduling system was built using Quartz.NET, an open-source framework that integrates seamlessly with. NET applications, automating the file detection and processing tasks efficiently. Once detected, the new data were promptly added to the database, ensuring a continuous and dynamic data integration process on either a weekly or monthly schedule, like the HL7 approach.

Given the diverse formats and labels of the data received from various sources, data processing and wrangling were required to standardize the information according to the SDRS PRRSV sequencing database’s specifications. The standardization process was implemented using the open-source Microsoft C# and. NET frameworks, chosen for their cross-platform compatibility, high performance, and scalability. The subsequent sections will detail these data processing and standardization procedures, which were essential for maintaining the integrity and consistency of the database.

### 2.2 Data processing

Data shared by different VDLs followed a different data structure organization due to VDLs’ specific customized data generation and data wrangling protocols to process submission forms, diagnostic testing, and testing results data. In certain instances, necessary metadata required for subsequent analysis of PRRSV ORF5 sequences were not present in the data collected from the VDLs. An inter-VDL data standardization measure was implemented to streamline the processing of received data before its integration into the server. Accordingly, a data standardization algorithm was implemented for metadata cleaning and processing. PRRSV ORF5 lineage classification using a reference set of sequences (20, 25) and variant classification using a machine learning algorithm (26) were implemented. Additionally, recognition of RFLP patterns, calculation of US commercially available PRRS modified live vaccine (MLV) nucleotide similarity, and determination of sequence length, presence and number of ambiguities using a customized algorithm written in C# and. NET were conducted. The subsequent subsections describe the specifics of data processing.

#### 2.2.1 Sequence metadata cleaning

The raw data collected from participant VDLs was characterized by diverse formats and included the PRRSV ORF5 sequences and related metadata such as site state, country, received date, specimen, farm type, Logical Observation Identifiers Names and Codes (LOINC) values, LOINC texts, Systematized Nomenclature of Medicine Clinical Terms (SNOMED CT) text, accession ID, and sample ID. For example, some VDLs recorded location information using full state names, while others employed abbreviations. Additionally, specimen discrepancies arose due to variations in how VDLs labeled the submitted specimens. For example, “Pool-Serum,” “Pooled Serum,” “BLOOD SERUM,” “Serum” and “Serum Pool” were different formats of specimens reported by the VDLs.

The sequences underwent a comprehensive pre-processing phase to ensure uniformity and consistency in the SDRS PRRSV sequencing database. This step involved standardizing the metadata to collate each ORF5 sequence with corresponding metadata at a sample ID level. Procedures for data standardization involved standardizing the states to a consistent two-letter state code format in the presence of state information or else labeling as “Unknown,” aligning the LOINC and SNOMED texts to their respective codes, and categorizing the specimen into consistent categories of nomenclature. The previous example of “Pool-Serum,” “Pooled Serum,” “Blood Serum,” “Serum,” and “Serum Pool” specimen labels was aggregated and labeled as “Serum” samples in the PRRSV sequencing database.

Additionally, to safeguard privacy and consistency, the sequences were VDL anonymized by assigning new sequence identification (ID) numbers. Each sequence was labeled with a unique ID comprising the term “SDRS” followed by its sequence of entry order in the database. Key metadata, including the original sequence accession ID, animal ID, and collated metadata attributes, were securely stored to enable traceability back to the originating VDL when necessary. This initiative standardized the IDs in the SDRS database and facilitated anonymization in any communication required between the participating VDLs. Sequences having greater than six ambiguities, a length lower than 597 nucleotides, and greater than 606 nucleotides were considered incomplete and not included in the analysis.

#### 2.2.2 Lineage and variant classification

Each PRRSV ORF5 sequence had a lineage classification assigned using ClassLog, a robust and versatile supervised machine learning application (29). ClassLog was selected for this project due to its exceptional speed, high accuracy, and seamless integration into high-throughput data processing pipelines (29). Additionally, ClassLog’s ability to decouple the classification process from the inference of viral evolutionary history (29) made it particularly well-suited for this project. A publicly available classification tool using the reference lineage dataset (20) has also been made available to end users through another work (30).

Briefly, ClassLog operates by accepting labeled data as input during the training phase, where each data point is associated with a specific classification label. The machine learning-based model learns from this labeled training data, enabling it to accurately classify new sequences into predefined categories based on the patterns it has recognized during training (29). Two classification models were trained to identify and assign the lineages to the PRRSV ORF5 sequences. For the first classification model, the training data comprised two sets of reference sequences, one corresponding to PRRSV-1 and the other to PRRSV-2. The PRRSV-2 training dataset included 32 labels belonging to PRRSV-2 lineages and sublineages (20), and one additional label was used to identify PRRSV-1, adding to a total of 1,287 PRRSV-1 and PRRSV-2 reference sequences. Once the model was trained, all newly collected ORF5 sequences were tested against the trained model to obtain their lineage classification labels. This approach ensured that the lineage assignments were accurate and consistent with the latest classification standards.

A second classification model was trained using an established PRRSV-1 lineage classification system (25). This model was trained on a reference set of 968 PRRSV-1 sequences, each assigned to one of the 18 distinct lineage labels. Sequences initially classified as PRRSV-1 by the first classification model were then analyzed using this secondary model to determine their specific lineage and sublineage. Maintaining separate lineage classification models for PRRSV-1 and PRRSV-2 provided a stepwise approach to first accurately identify PRRSV-1 sequences and subsequently classify them into the correct lineage and sublineage.

ORF5 sequences were also classified into genetic variants using a fine-scale system to group closely related sequences under the same variant label (26). The variant classification is a dynamic fine-scale classification scheme within the lineage system that provides better resolution to the relatedness of PRRSV-2 viruses (26). This classification relies on a pre-trained random forest machine learning algorithm that assigns variant IDs to new sequences (26) as they enter the SDRS database. The model, developed by external researchers, is updated quarterly to reflect new data and trends. At SDRS, the implementation was customized to maintain local copies of the model provided by the variant classification authors (26, 31), with an automated process in place to check for updates each quarter, ensuring the model remains current and accurate, hence maintaining the accuracy of variant IDs assigned to sequences. A webtool version of this model is also available for end users (31).

#### 2.2.3 Restriction fragment length polymorphism (RFLP) pattern analysis

Another historically widely utilized method for PRRSV-2 classification in North America is based on RFLPs (23). This approach uses a set of restriction enzymes, *MluI, HincII*, and *SacII* in that order to discern unique cut patterns within the ORF5 sequence. Each enzyme generates a distinct cut pattern. These patterns are translated into numeric codes, providing a standardized classification system for PRRSV-2 (23). Traditionally, RFLP classification required a gel-based PCR assay to visualize the cut patterns. However, with advancements in computational methods, RFLP patterns can now be predicted directly from ORF5 sequencing data. Even though new RFLP patterns are no longer assigned, the usage of the terminology is still very popular, requiring such implementation.

In the SDRS PRRSV sequencing database, every ORF5 sequence underwent RFLP pattern calculation as part of the data processing workflow. To handle the complexity of calculations and large data volumes, we parallelized the RFLP analysis process, significantly reducing time complexity and enabling efficient processing of the high number of sequences. An algorithm for RFLP pattern calculation was written in C# programming language.

The process began by aligning each sequence to an assigned reference sequence in the database to ensure accurate identification of restriction sites. Alignment accounted for sequence length variations, ensuring cut site identification consistency. Once aligned, the sequence was then scanned for the specific nucleotide motifs of the restriction enzymes *MluI* (ACGCGT), *HincII* (GTYRAC), and *SacII* (CCGCGG), including their reverse complements, to identify the cut sites. For *HincII*, the “Y” represents “T” or “C” and “R” represents “A” or “G” nucleotides, allowing for four possible combinations: GTCAAC, GTCGAC, GTTAAC, and GTTGAC, which, along with their reverse complements, were used to identify the cut sites. The positions of these cut sites within the ORF5 sequence determined the RFLP by looking up combinations of positions in a static code look-up table for each enzyme. The look-up table was the same as defined by industry and research standards (32), which was maintained by the UMN VDL and discontinued in 2024 (Rossow S., personal communication). The resultant hyphenated values, in order of *MluI, HincII and SacII*, were then stored in the database alongside the individual sequences. An ORF5 RFLP has not been established for PRRSV-1 sequences, and PRRSV-1 ORF5 sequences were marked as PRRSV-1.

#### 2.2.4 Vaccine similarity calculation

The calculation of vaccine similarity is vital for understanding the potential evolutionary origins of circulating virus strains. To streamline and expedite this process, parallelization techniques were used, allowing for faster and more efficient analysis of large datasets. Presently, there are five commercially available PRRSV-2 vaccines in the US: Ingelvac PRRS^®^ MLV (GenBank AF066183), Prime Pac^®^ PRRS (GenBank DQ779791), Fostera^®^ PRRS (GenBank AF494042), Prevacent^®^ PRRS (GenBank KU131568), and the relatively recent PRRSGard^®^. Ingelvac PRRS® ATP (GenBank DQ988080) MLV was also included, although it is no longer commercially available in the US. The SDRS PRRSV sequencing database meticulously calculated and recorded the percentage homology of each ORF5 sequence against these vaccine strains to monitor the genetic relationship between circulating virus strains and the vaccines. To achieve this, each PRRSV-2 sequence in the database was first aligned with each vaccine strain using MAFFT, a widely used multiple sequence alignment program (33). MAFFT ensures accurate alignment, allowing for precise comparison of sequences. Following alignment, the Hamming distance (34), which measures the number of differences between two sequences, was calculated between the vaccine strain and each individual sequence. This distance was then converted into a percentage similarity value, providing a quantifiable measure of homology. The database included specific columns to store these percentage similarity values, facilitating easy retrieval and analysis when needed.

Furthermore, the database also documented whether a sequence exhibited characteristics of a vaccine strain or a wild-type strain. Any PRRSV-2 sequence demonstrating equal to or greater than 99% homology with any of the PRRSV-2 vaccine strains was categorized as a vaccine-like sequence, indicating a close genetic relationship with a known vaccine strain. The 99% or greater homology identity threshold was selected as a conservative cutoff to differentiate wild-type and vaccine-derived sequences. This threshold was chosen based on the expectation that viruses directly derived from vaccine strains should exhibit minimal genetic divergence, typically within 1% variation, similar to sequences retrievable from a vaccine bottle. In the Sanger sequencing process, up to 6 nucleotides or 1% genetic difference can be due to errors in the process (35). Genetic differences of >1% from field samples and the MLV product recovered from a bottle would indicate genetic changes during replication. Conversely, sequences showing over 80% homology with Lelystad, a PRRSV-1 referent virus (GenBank M96262) were designated as wild-type European sequences. All other sequences were classified as wild-type sequences.

#### 2.2.5 Sequence ambiguity count and length calculation

The length of each sequence and the number of ambiguities were documented as essential metadata to evaluate the quality and completeness of the sequences. Sequence length was defined as the total number of nucleotides in the sequence, while ambiguities referred to the non-A, C, G, or T characters within the sequence. An algorithm was written using C# programming language to treat sequences as strings, counting the number of characters and identifying any ambiguous nucleotides. This process allowed for the accurate assessment of sequence integrity, with ambiguities, which typically arise from consensus issues during Sanger sequencing, minimized to improve data reliability. Additionally, by recording the length of each sequence, it was ensured that only complete ORF5 sequences, typically 603 nucleotides (PRRSV-2) and 606 nucleotides (PRRSV-1), were considered in the analyses of novel sequences. This step provided a path for filtering out incomplete sequences that could potentially skew the identification of novel sequences. The incomplete sequences were added to the database for future reference but were excluded from the analysis.

#### 2.2.6 Temporal trends of PRRSV ORF5 detection

A comprehensive temporal analysis, using Python 3 programming language and SQL queries, assessed the distribution of PRRSV sequences received across different years and months. SQL queries were used to filter complete sequences with fewer than six ambiguities (35), excluding all the other sequences that did not meet the criteria. When more than five ambiguities are present in a PRRSV ORF5 sequence recovered by Sanger the sequence homology or phylogenetic analysis should be cautiously interpreted (35). This enabled tracking the number of sequences received each year, breaking them down by swine age groups, location, and the specimen from which the samples originated. To simplify the data analysis, specific categories were consolidated into broader groups. The finishing and nursery categories were combined under the grow-finish production phase, while the adult, breeding herd, replacement, boar stud, and suckling piglet categories were merged into the adult/sow phase. The analysis also delved into the detection of PRRSV-2 and PRRV-1 lineages and PRRSV-2 variants, offering a clearer picture of the evolutionary dynamics of the virus. The purpose of studying the temporal trends of lineages was to identify lineages that were more active and prone to significant nucleotide changes over time, potentially leading to novel sequences. Similarly, variant trends were monitored to determine if emerging variants signaled the development of new strains within specific lineages. Additionally, RFLP patterns were scrutinized to pinpoint common combinations of cut sites, providing a broader view of how frequently these patterns appeared in PRRSV sequences. PRRSV-1 sequences were excluded from variants and RFLP analysis.

#### 2.2.7 Novel sequence identification

To effectively identify novel PRRSV sequences potentially circulating in the field, a specialized instance of the NCBI Basic Local Alignment and Search Tool (BLAST) software (36) was implemented in SDRS data processing steps. This proprietary instance was tailored to create a PRRSV BLAST database using PRRSV ORF5 nucleotide sequence data from the PRRSV sequencing database, customized for sequence searches. The BLAST database is securely hosted on the scalable server infrastructure of the SDRS server housed in the ISU Veterinary Diagnostic and Production Animal Medicine Department, ensuring the robustness and integrity of data.

An algorithm was designed to identify novel sequences within the PRRSV sequencing database systematically. The initial BLAST database was populated with ORF5 sequences collected between January 1^st^, 2006, and December 31^st^, 2009. From January 1st, 2010, to December 31st, 2024, onwards, new sequences were analyzed to determine whether they were novel. Data between January 1st, 2006, and December 31st, 2009, were used as a baseline to build the BLAST database. For each PRRSV ORF5 sequence added to the SDRS database after this date, known as the query sequence, the algorithm would first retrieve all sequences from the SDRS PRRSV sequencing database and BLAST the query sequence to the database that had been recorded before the query sequence’s received date. The received date was the one reported by the participant VDLs. This approach ensured that only sequences available prior to the query were considered for comparison, maintaining accuracy in identifying novel sequences. A nucleotide BLAST search was then performed on this subset of sequences to determine the one with the highest percentage similarity to the query sequence. The sequence ID and corresponding percentage similarity of the closest sequence match were recorded in dedicated columns within the PRRSV sequencing database. After this comparison, the query sequence was integrated into the BLAST database, ensuring that the database remained up to date with the most recent data.

Currently, each sequence added to the SDRS PRRSV sequencing database undergoes this collation process, ensuring that newly emergent sequences are promptly and accurately identified. The identification process is designed to account for sequences with received dates before the query sequence’s received date, ensuring that comparisons are only made against sequences logged before the query sequence, reducing computational complexity, and ensuring accurate results. To accommodate the receiving of sequences with retrospective receiving dates, a process was designed to identify the oldest received date on the incoming data and re-run all the steps again for that day and afterward. As an example, if on January 31st of 2023, a sequence was entered as a received date in the VDL as December 31st, 2022, the process will run again starting on December 31st, 2022. Given the computational demands of this process, particularly with large datasets, parallelization was used to accelerate the BLAST searches. This approach significantly reduced the time required for each search, enhancing the overall efficiency of the algorithm. Analysis of novel sequences was performed using Python 3 programming language on a Windows-based system with 16 GB RAM processor, ensuring sufficient computational power to handle the data volume and complexity.

Sequences that exhibited less than 95% similarity to the closest existing sequence, contained six or fewer ambiguities, and had a length of 597 nucleotides or more were categorized as novel sequences. The 95% similarity threshold was chosen to account for the high homology typical of PRRSV, while the ambiguity limit and length criteria ensured that only high-quality, complete sequences were flagged as novel. A 95% similarity threshold is a very conservative one, but any cut-off level can be used for new sequence identification since the similarity levels are registered in the database. After ensuring that the databases were fully populated with all necessary metadata and capable of dynamically handling new data entries, the analysis step was started. The primary focus was on identifying and characterizing new PRRSV sequences. Further, detailed data analytics was conducted to assess the distribution of novel sequences across different farm types, temporal patterns, geographic regions (such as specific states), lineages, variants, and RFLP patterns. The analysis also examined the formation of homologous groups of novel sequences, which could pose significant threats to swine production systems if left unaddressed. The frequency of novel sequence detection was also summarized by site state and aimed to identify geographic regions with a higher likelihood of detecting novel sequences, comparing these trends with the number of positive PRRSV cases submitted from each state. This analysis aimed to understand regional patterns in PRRSV evolution and potential hotspots for new variants by farm type. Novel sequences were also characterized according to lineage, variants, and RFLP patterns. To track the frequency of novel sequences in the database, the occurrence ratio of novel sequences to overall (count of total) sequences was calculated and defined as novel sequences per one thousand sequences.

Finally, sequence persistence analysis was performed using the BLAST database built from sequences in the PRRSV sequencing database. The objective was to monitor novel sequences and determine if they formed distinct detection expanding groups. A detection expanding group was defined as recovery of a set of at least 20 sequences since the initial novel detection, with a sample receipt date after the novel sequence, exhibiting more than 98% similarity to the novel sequence. This threshold was set based on a prior study that defined a minimum of 15 PRRSV sequences to classify as a new group (37), ensuring that the identified groups were robust and significant.

### 2.3 Implementation of a public external SDRS BLAST tool

The BLAST database, initially developed to identify novel sequences within the SDRS PRRSV sequencing database, was further utilized to create a web-based platform that allows users to provide a given PRRSV ORF5 sequence and search for sequences present in the database similar to the given query sequence. This web application was built using C# and the. NET framework, providing a robust and scalable environment for sequence search. It integrates directly with the PRRSV BLAST database, enabling real-time sequence searching.

A customized web application provides users with an input query box on the SDRS website, where they can submit a PRRSV ORF5 nucleotide sequence of interest (the query sequence) along with a custom sequence name for reference. The platform utilizes a heuristic approach based on the BLAST algorithm, which approximates the Smith-Waterman algorithm (36, 38) to identify sequences with the highest similarity to the query. This method allows for efficient searching while maintaining accuracy in sequence alignment. The algorithm identifies similar sequences within the BLAST database and the percentage of nucleotide identity, i.e., similarity. Once the BLAST algorithm identifies the top matching sequences, the platform retrieves relevant metadata associated with the PRRSV sequence in the SDRS database using the unique SDRS sequence identifier SDRS ID. Retrieved metadata includes the sequence’s received date, geographic location, i.e., state, lineage, variant, RFLP pattern, and the nucleotide identity. Users can customize the number of responses to display from one to 50 top matches. This user-friendly interface enables researchers, veterinarians, and stakeholders to quickly access crucial information for comparative analysis.

## 3 Results

The analysis was conducted using the SDRS PRRSV sequencing database, which comprised a total of 115,643 PRRSV ORF5 sequences received between January 1st, 2006, to December 31st, 2024. Out of the 115,643 sequences received, 731 were excluded from the analysis for not meeting the criteria for complete sequences, incomplete sequences being defined as sequences having: greater than six ambiguities, a length lower than 597 nucleotides, and greater than 606 nucleotides. Of the 731 incomplete sequences, 599 sequences had greater than six ambiguities, two sequences were longer than 606 nucleotides, and 130 sequences were shorter than 597 nucleotides. This brought the total number of complete sequences to 114,912.

**Figure 2:**
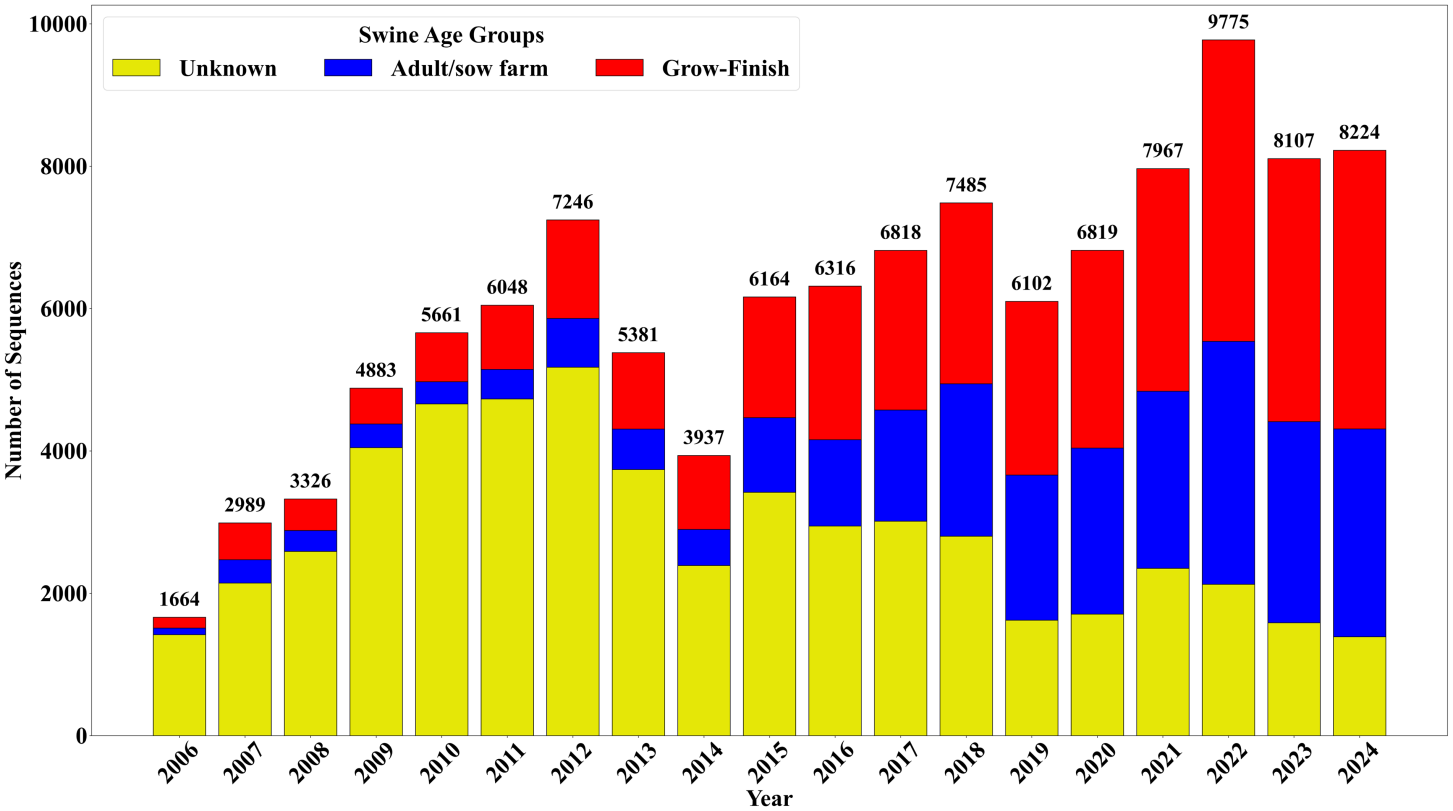
Swine age group distribution of complete sequences received between January 2006 and December 2024.

Of the 114,912 complete sequences received, 46.87% (*n* = 53,855) lacked associated swine age groups data. Sequences from adult/sow farms accounted for 22.22% (*n* = 25,529) entries, while grow-finish farms contributed 30.91% (*n* = 35,528) sequences (Figure 2). A further breakdown of the adult/sow category, which was created by consolidating multiple farm types, is as follows: suckling piglet 54.62% (*n* = 13,939), breeding herd 17.49% (*n* = 4,463), replacement 14.65% (n = 3,740), adults 13.11% (*n* = 3,347), and boar studs 0.16% (*n* = 40). Of the 35,528 sequences from grow-finish farms, 55.52% (*n* = 19,725) came from finishing and 44.48% (*n* = 15,803) from nursery farms.

Between January 2020 and December 2024, 40,892 sequences were added to the SDRS PRRSV sequencing database. Sequences from adult/sow farms represented 34.19% (*n* = 13,983), while grow-finish farms contributed 43.41% (*n* = 17,750) (Figure 2). In this period an average of 681 sequences were received per month, marking an increase of 241 sequences over the monthly average of 440 sequences recorded between January 2006 and December 2019. Notably, sequences categorized under the unknown farm type category dropped to 22.40% (*n* = 9,159), a significant reduction compared to the 60.38% (*n* = 44,696) recorded from 2006 to 2019, and representing an improvement in swine age group identification than in previous years. This reflects a consistent decrease in the percentage of sequences from unknown farms, alongside a marked identification of submissions from grow-finish over time.

**Figure 3:**
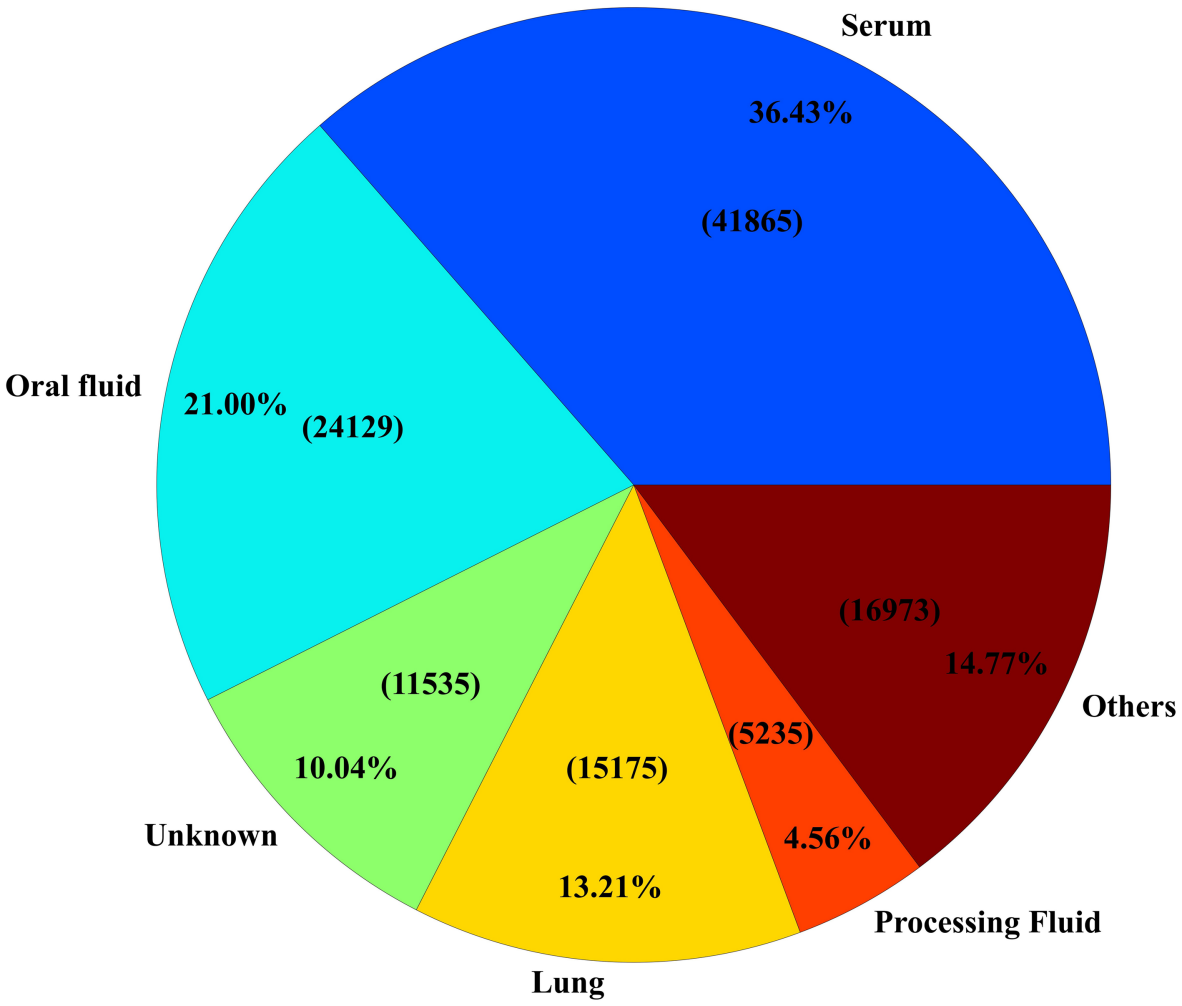
Specimen distribution of samples received. The ‘Others’ section consists of all specimen types which were received less than 5,000 times.

Among all specimens used for ORF5 sequencing, serum samples accounted for the largest share, representing 36.43% (*n* = 41,865) of specimens (Figure 3). Serum was followed by oral fluid and lung samples, which made up 20.99% (*n* = 24,129) and 13.21% (*n* = 15,175) of the sequences, respectively (Figure 3). Notably, 10.04% (*n* = 11,537) of samples lacked associated specimen information. Within the oral fluid samples, 44.27% (*n* = 10,683) were reported from grow-finish farms, while 30.41% (*n* = 12,730) of the serum samples came from adult/sow farms. A notable increase in oral fluid and processing fluid usage for PRRSV ORF5 sequences occurred after 2009 and 2017, respectively, coinciding with the description and validation of these sample types for PRRSV monitoring and surveillance (10, 39–42).

Lineage is an important parameter for understanding the evolutionary dynamics of PRRSV. Implementation of the recently updated PRRSV-2 lineage classification system (20) led to a reduction in the percentage of unclassified/unknown sequence lineages from 10.12%, using the previously proposed classification system (12, 21, 24), to 1.92% across all sequences in the database. The trends in PRRSV lineage distribution between January 2006 and December 2024 revealed useful insights. Of the total number of complete sequences analyzed, 77.89% (*n* = 89,516) were classified as wild-type sequences, indicating a significant majority of the recovered PRRSV strains. Only 1.97% (n = 2,266) of the sequences were identified as wild-type PRRSV-1 (European) sequences, representing a small proportion of PRRSV-1 strains in the dataset. The remaining 20.14% (*n* = 23,136) were categorized as modified live vaccine-like sequences, highlighting the presence of vaccine-derived strains that may be circulating in the field. Sublineage 1D (L1D) had always been present in the database but gained traction since 2019 with the introduction of an L1D vaccine in the US, i.e., Prevacent^®^ PRRS, representing 7% (*n* = 2,865) of sequences being detected from 2020 to 2024. Sequences classified as Ingelvac ATP-like were rarely detected after 2020, and Ingelvac MLV-like (*n* = 14,911) represented 12.98% of detections.

**Figure 4:**
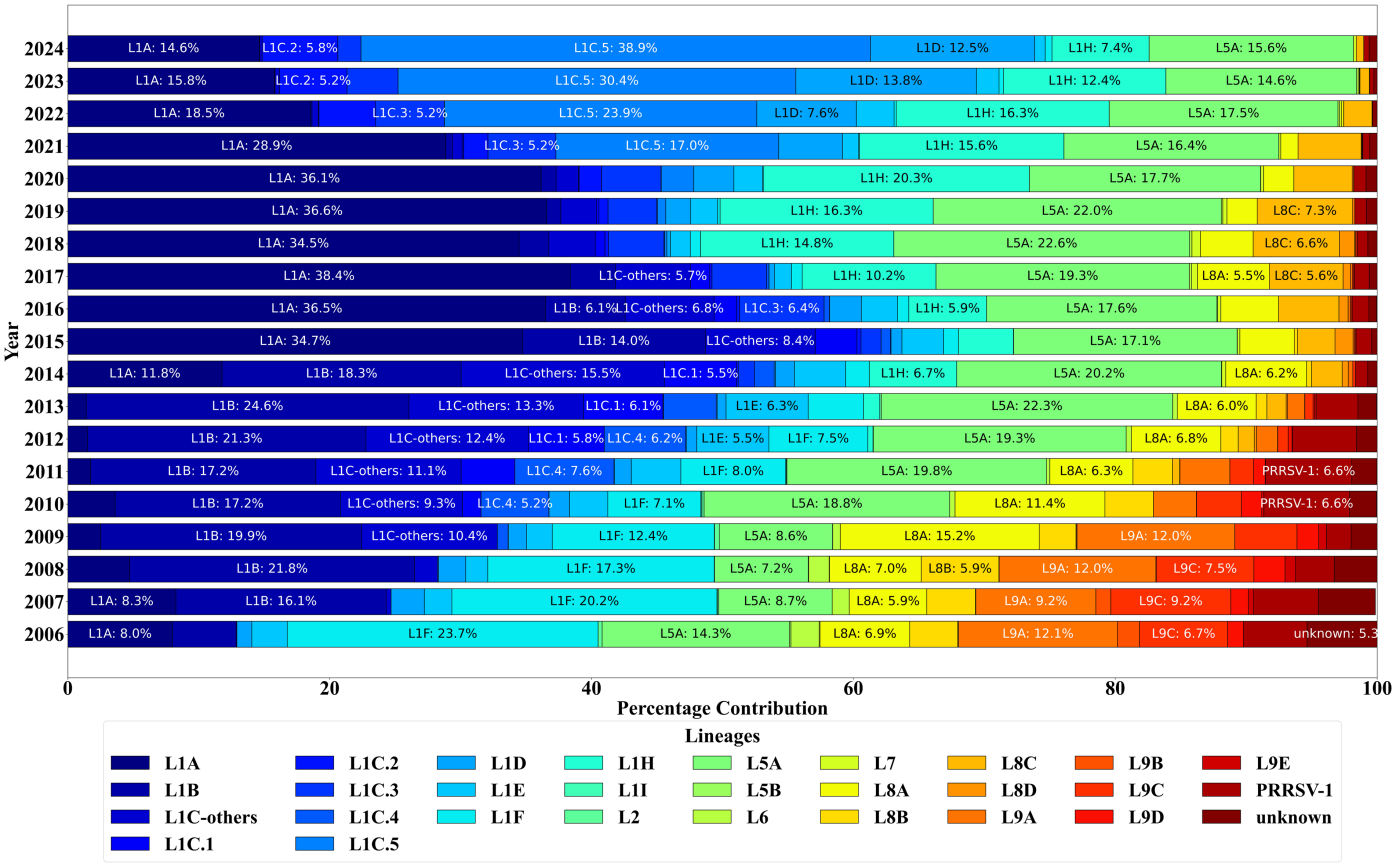
PRRSV ORF5 sequence detections by lineage classification between January 2006 and December 2024.

Sublineage 1A (L1A) (wild-type) was the most detected PRRSV-2, representing 19.63% (*n* = 22,553) of the sequences, followed closely by sublineage 5A (L5A) (vaccine-like), which accounted for 17.42% (*n* = 20,012) (Figure 4). Notably, the L1A sequences spiked in detection in 2014, were the predominant sequences detected until 2020, and have been waning since 2020. Sublineages 1C.2 and 1C.3 (wild types), which first emerged in 2013 and 2014, have shown a steady increase in the number of sequences detected after 2020. The first detection of sublineage 1C.5 dated from 2009, with less than five detections until 2018. Sublineage 1C.5 frequency of detection increased starting in 2020, becoming the predominant sublineage in 2024 with 39% of detections. Nevertheless, during 2024, sublineage 1C.2 was the major wild-type detected in Illinois, the second in Iowa, and the third in Minnesota, demonstrating regional expansion and increased epidemiological importance of this sublineage. In contrast, sublineage 1C.1 and sublineage 8D (L8D) have consistently declined, with L8D having zero detections since 2019. The sublineages within lineages 8 (L8) and 9 (L9) have shown a decline in detections, with only L8C being detected in 2024 among all the L8 sublineages. Over the years, there has been a noticeable decrease in the number of sequences classified as ‘unknown’ lineages, reflecting improved classification accuracy. Lineage 1B (L1B) was predominantly detected before 2014 but was rarely detected in 2023, with four detections, and one detection in 2024.

Providing correct information in the submission forms for VDL testing is a crucial first step in epidemiological investigations. Three of the L8D sequences in the SDRS database had a site state informed as Minnesota, while all other sequences were identified as received from Mexico. Since no circulation of L8D sequences has been reported in the US, this will likely be an error when informing the site state in the submission forms. In this study, four ORF5 sequences were identified as L8E. The L8E sequences are mainly HP-PRRSV strains initially identified in China in 2006 (43, 44) with no circulation of this viral strain in the US (20). Additionally, one sequence for lineage 3 (L3), three sequences for lineage 4 (L4), and six for L11 were reported without evidence of the presence of these sequences circulating in the field in the US. The presence of L3, L4, L8E, and L11 sequences in this study is likely due to research cases not identified as “research” during the submission process and being shared with the SDRS project without representing field clinical cases.

Within the PRRSV-1 sequences, 57.50% (*n* = 1,303) were classified as L1.1A, 25.24% (*n* = 572) as L1.1B, 15.18% (*n* = 344) as L1.1C, and 1.19% (*n* = 27) as L1.1 sequences. Only two PRRSV-1 sequences were classified as “unknown” lineage within PRRSV-1. PRRSV-1 is still present in the US at a lower detection level when compared with PRRV-2, with 26 detections in 2023 and 34 in 2024 coming from Iowa, Indiana, North Carolina, Pennsylvania, and Utah.

Across 172 PRRSV-2 available variants classifications (26), 165 were detected in this work. Variant classification results showed that the most detected variant was 5A.1, representing 17.22% (*n* = 19,788) of all sequences, followed by 1A-unclassified at 12.07% (*n* = 13,875) and 1C.5 at 9.42% (*n* = 10,822). Among these, 33.65% (*n* = 6,658) of sequences with 5A.1 variant, 22.42% (n = 3,111) of sequences with 1A-unclassified variant, and 87.49% (*n* = 9,468) of sequences with 1C.5 variant was identified between January 2020 and December 2024. Another 26.57% (*n* = 30,529) of sequences were labeled as “unclassified,” meaning they had a defined lineage but were not assigned a specific variant. Only 12.59% (*n* = 3,843) of these unclassified sequences were recorded between January 2020 and December 2024. The “undetermined” variant label was assigned to 2.73% (*n* = 3,137) of the sequences, with 31.34% (*n* = 983) of them occurring during the same recent period. The majority of sequences, 68.21% (*n* = 78,384), were successfully assigned both a lineage and a variant label.

RFLP analysis served as another identifier of PRRSV-2 sequence megatrends of detection and encompassed 309 unique RFLP patterns, which also included the PRRSV-1 strain designation. The ten most frequently detected PRRSV-2 RFLP patterns represented 80.93% (*n* = 93,003) of the sequences and were represented by: 1–4-4, 2–5-2, 1–8-4, 1–7-4, 1–4-2, 1–3-2, 1–3-4, 1–26-2, 1–18–2 and 1–2-4 patterns. The remaining 19.07% (*n* = 21,909) of sequences are represented by the remaining 299 RFLP patterns.

Iowa contributed the highest number of sequences with 29.39% (*n* = 33,770), followed by Minnesota 18.07% (*n* = 20,761), Missouri 4.85% (*n* = 5,573), North Carolina 4.73% (*n* = 5,439), Illinois 4.07% (*n* = 4,675), Oklahoma 3.8% (*n* = 4,372), Indiana 3.27% (*n* = 3,753), Nebraska 2.66% (*n* = 3,053), Colorado 1.57% (*n* = 1,802), and Texas 1.33% (*n* = 1,530). Additionally, 19.72% (*n* = 22,665) of sequences had no site state location data, with 94.21% (*n* = 21,352) of these reported between January 2006 and December 2019.

**Figure 5:**
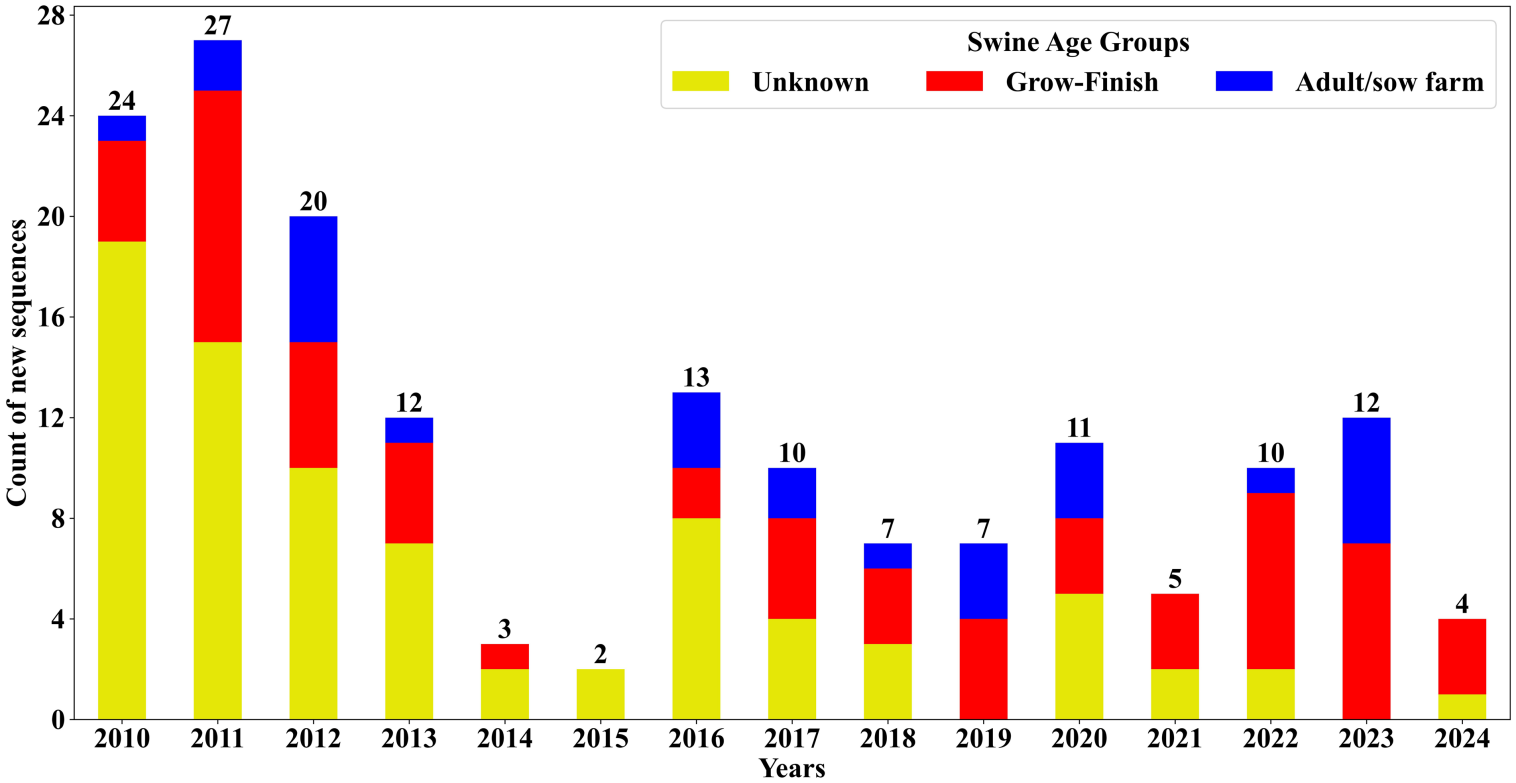
Novel PRRSV ORF5 sequences identified and distributed by swine age group from January 2010 to December 2024. The number of novel sequences classified as ‘unknown’ swine age group has decreased over the years.

The data analysis for identifying novel sequences was conducted on records spanning from January 2010 to December 2024 using 102,050 complete sequences within this period and identified 167 novel sequences. Subsequently, the metadata of these sequences was analyzed to recognize any discernable patterns in virus evolution and spread. The first investigation assessed the detection of novel sequence detections across different farm types: adult/sow, grow-finish, and unknown. Of the 167 newly identified sequences, the majority, constituting 47.9% (*n* = 80), were classified under the “unknown” category as they did not have any swine age group information. This was followed by 35.93% (*n* = 60) identified in grow-finish farms and 16.17% (*n* = 27) in adult/sow farms. Among the novel sequences from grow-finish farms, 53.33% (*n* = 32) originated from finishing and 46.67% (*n* = 28) were from nursery. Similarly, within the 27 novel sequences detected in adult/sow farms, 77.78% (*n* = 21) were from suckling piglets, while 11.11% (*n* = 3) came from both adult and breeding herd farms combined. Over the period between January 2020 to December 2024, 42 novel sequences were detected, representing 25.15% (*n* = 42) of the total novel sequence identifications. Of these, ten sequences lacked farm type information, while nine and 23 sequences were associated with adult/sow and grow-finish categories, respectively. Notably, 38.98% (*n* = 23) of the novel sequences categorized under the grow-finish farm type were detected within the last 5 years (January 2020 to December 2024). Figure 5 illustrates the distribution of farm types among the novel sequences.

**Figure 6:**
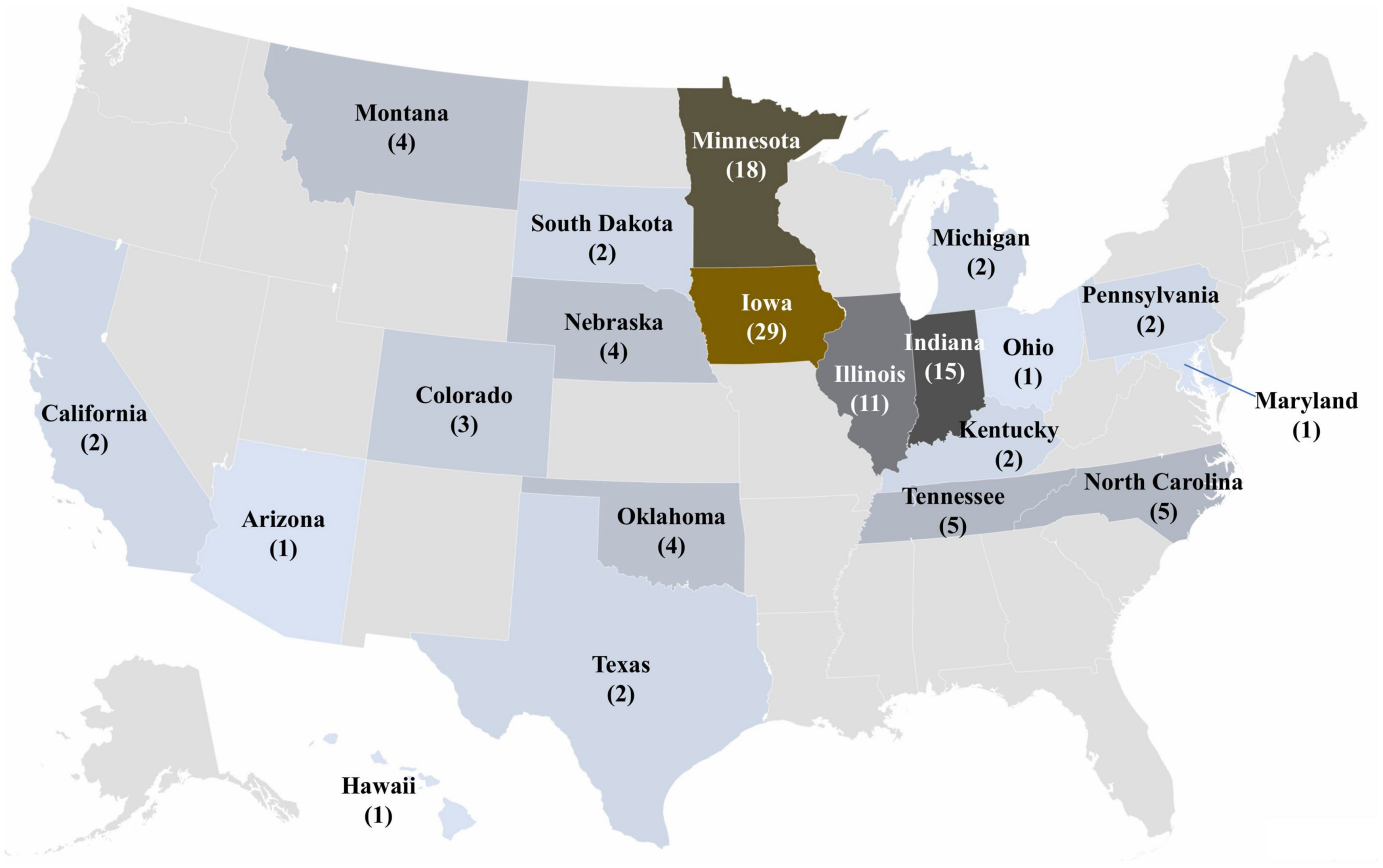
Distribution of novel sequences between January 2010 and December 2024. 53 of the novel sequences had no state information and were labeled as “unknown” locations.

Among the 42 states represented in the SDRS PRRSV database, 20 states had the detection of novel sequences. Notably, 31.74% (*n* = 53) of the 167 novel sequences lacked state information. Sequences recovered from Iowa sites accounted for the highest proportion of novel sequences at 17.37% (*n* = 29), followed by Minnesota 10.78% (*n* = 18), Indiana 8.98% (*n* = 15), and Illinois 6.59% (*n* = 11). The remaining 41 sequences were distributed across 16 states, with each state contributing between 1 and 5 sequences (Figure 6).

**Figure 7:**
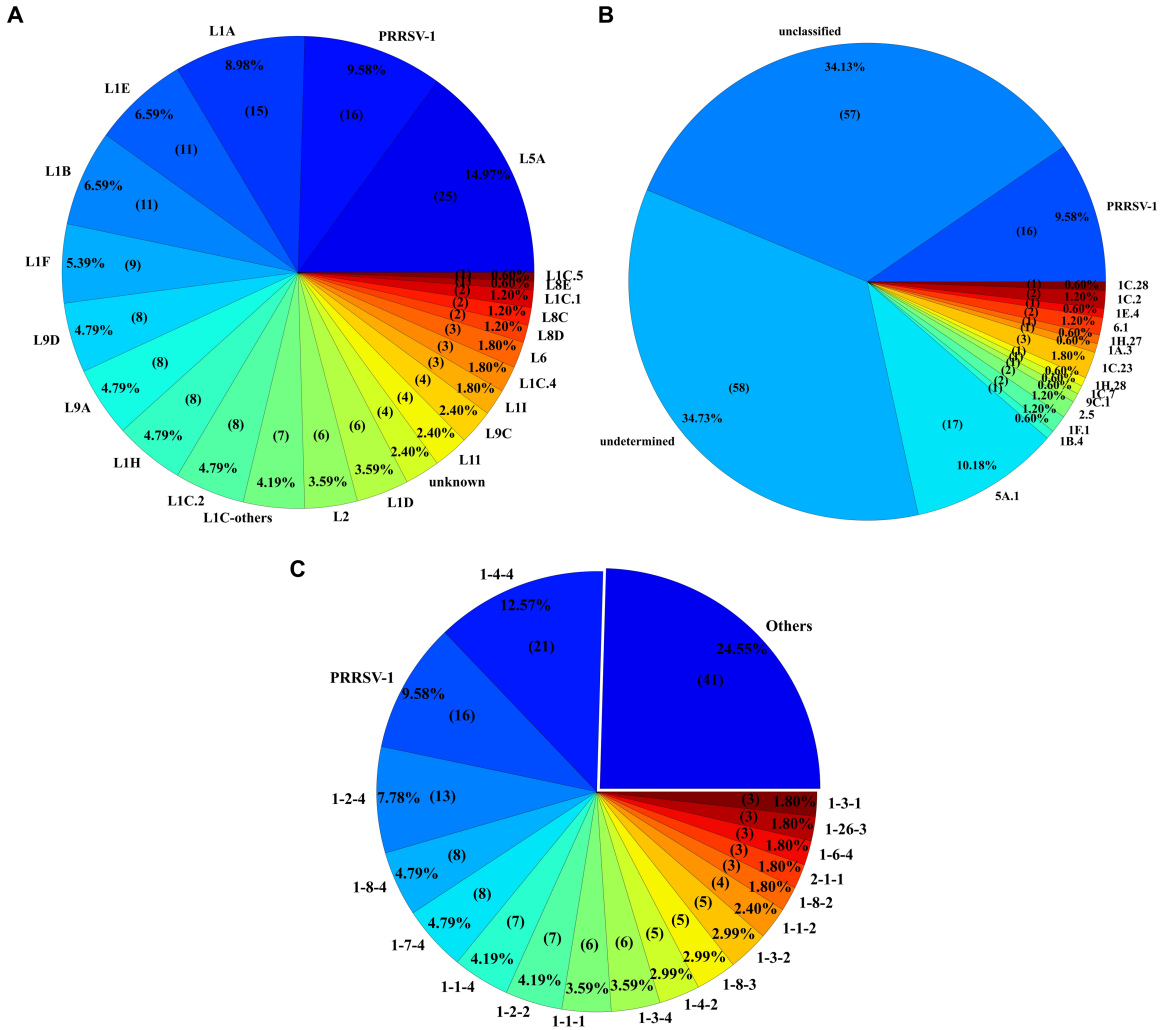
**(A)** Illustration of the lineage or sublineage classification for 167 novel sequences. Sequences were assigned as novel if they had less than or equal to 6 ambiguities and length between 597 and 606 nucleotides. **(B)** Variant classification of the novel sequences with all unclassified sequences included under the ‘unclassified’ variant label. **(C)** Most frequent RFLP pattern seen in the novel sequences. The ‘Others’ section in the pie chart reflects the RFLP patterns seen twice or less.

Among the novel identified sequences, 14.97% (*n* = 25) were classified as L5A, while another 9.58% (*n* = 16) were designated PRRSV-1. Notably, 2.39% (*n* = 4) of the sequences remained classified as unknown. Additionally, L1A, characterized by the high number of sequences, contributed 8.98% (*n* = 15) of the 167 novel sequences, followed by L1B and sublineage 1E (L1E) with 6.59% (*n* = 11) novel sequences each. A multitude of other lineages and subgroups also contributed to the novel sequences, as depicted in Figure 7A. Of the 167 novel sequences, 34.73% (*n* = 58) were labeled as “undetermined” and 34.13% (*n* = 57) as “unclassified” variants. The remaining 52 sequences were classified with specific variant numbers within their respective lineages. 15.56% (*n* = 26) sequences were classified as 1C-unclassified variant followed by 10.18% (*n* = 17) sequences being classified as 5A.1 variant (Figure 7B). Among the 16 PRRSV-1 novel sequences, six sequences belonged to the L1.1 PRRSV-1 sublineage, followed by four L1.1A and two L1-unclassified sequences, respectively.

RFLP pattern 1–4-4 12.57% (*n* = 21) and PRRSV-1 9.58% (*n* = 16) accounted for the highest proportions of the 167 novel sequences. Noteworthy, frequently the RFLP patterns also included 1–2-4 (*n* = 13), 1–8-4 and 1–7-4 (*n* = 8 each), 1–1-4 and 1–2-2 (n = 7 each), and 1–3-4 and 1–1-1 (*n* = 6 each). The remaining 44.91% (*n* = 75) of sequences exhibited one of the additional 38 RFLP patterns, each appearing fewer than 6 times in the analysis. Figure 7C delineates the distribution of RFLP patterns among the novel sequences.

**Figure 8:**
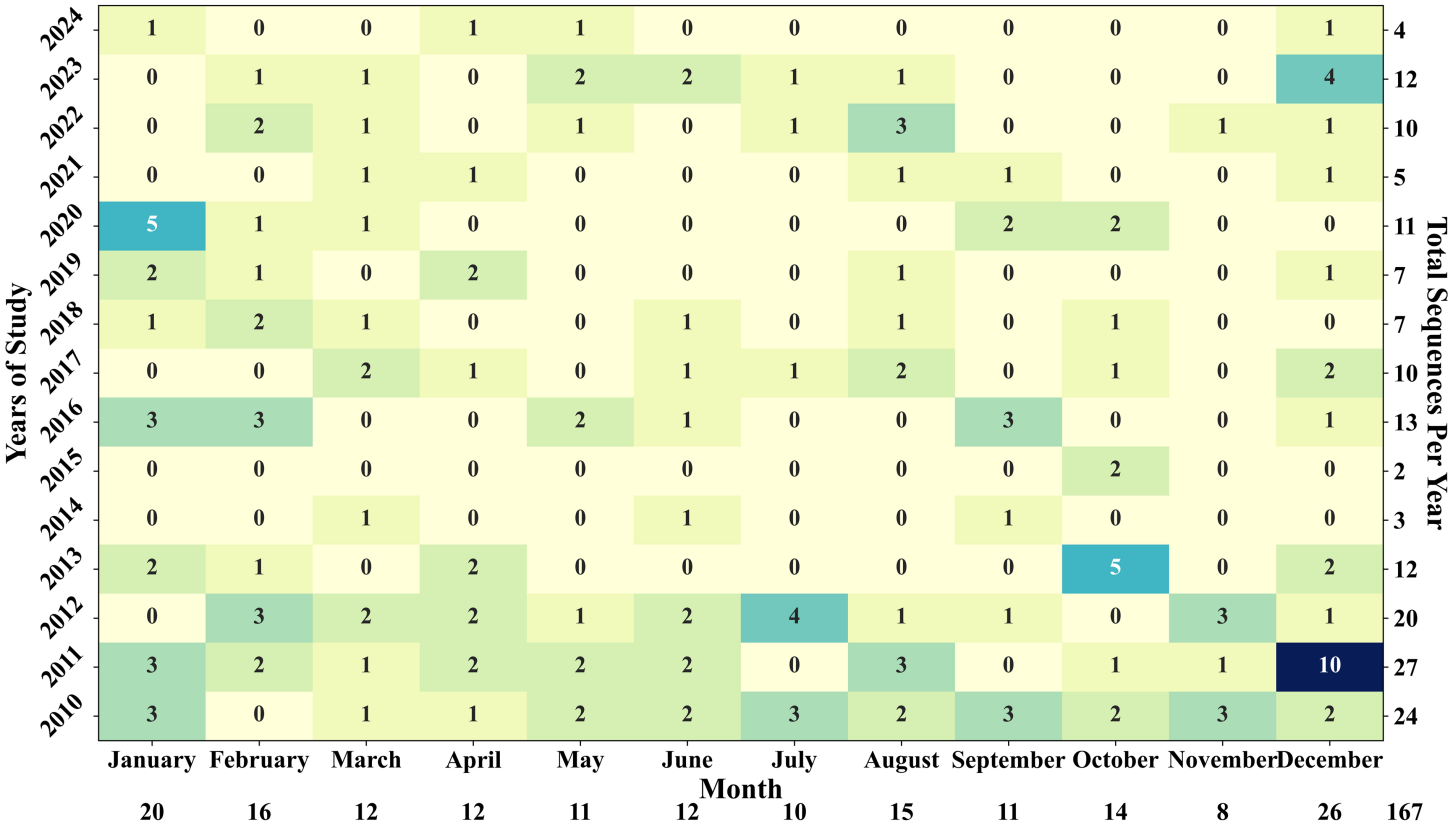
Yearly and monthly number of novel sequence detections displayed in a heatmap. December 2011 had the highest number of detections recorded, followed closely by October 2013 and January 2020, with 5 detections each.

Seasonal variation in PRRSV sequence detection was also observed, with the frequency of novel sequence detections fluctuating throughout the year. December and January emerged as the months with the highest number of novel sequence detections since 2010, with 26 and 20 sequences detected, respectively (Figure 8). The summer months, spanning from June to September, witnessed an average of 14 novel sequences detected, with the highest detection of 4 sequences in July 2012. Notably, 2011 saw the highest number of novel sequences detected at 27, while 2015 recorded the lowest at only 2 novel sequences. Figure 8 provides a comprehensive overview of sequence detections across each month within the studied years. For the 102,050 sequences analyzed, a novel sequence was detected on average every 611 sequences. January and December had high sequence submissions, with 9,790 and 11,367 sequences received, respectively, yielding an average detection of a novel sequence for every 489 sequences in January and 437 in December. Interestingly, August, with 6,179 sequence submissions, showed the shortest gap, with a novel sequence identified for every 411 sequences. In contrast, November had the longest gap, with 10,882 sequences received between 2010 and 2024 but only eight novel sequences detected, meaning a novel sequence was identified only once every 1,360 sequences during that month.

A pivotal analysis aspect involved identifying sequences in the database and tracing the novel sequences’ evolutionary trajectory, determining if they formed distinct groups after the initial detection and characterization as a novel sequence. Among the 167 novel detected sequences, a significant portion, 52.69% (*n* = 88), appeared as singular instances in the database, without any counterparts with 98% or greater similarity. Additionally, 43.71% (*n* = 73) of the novel sequences exhibited between one to ten occurrences of highly similar sequences. Another 1.8% (*n* = 3) of the novel sequences had between ten to 20 instances of detection since their initial appearance (Table 1). Only three novel sequences demonstrated more than twenty similar sequences each, sharing 98% or greater similarity with the original novel sequence (Table 1).

**Table 1:** Breakdown for novel sequences with 10 or greater similar sequences detected with 98% or greater similarity to the original novel sequences.

Masked Sequence ID	Received Date	Location	Lineage	RFLP	Variant	Number of detections after emergence
Seq1	3/23/2018	OK	L1H	1–12–4	1H.28	>49
Seq2	6/16/2010	IA	L1C-others	1–13–2	L1C-unclassified	23
Seq3	11/8/2010	IA	L1B	1–18–2	1B.4	>49
Seq4	12/7/2010	MN	L9D	1–4-1	undetermined	17
Seq5	1/1/2011	Unknown	L1I	1–8-2	undetermined	12
Seq6	6/6/2018	MI	L9D	1–1-1	undetermined	10

The BLAST database, originally created to identify novel PRRSV sequences, was enhanced into a web-based tool (SDRS BLAST tool, https://fieldepi.org/sdrs/blast-tool/) with a user-friendly interface. Accessible through the SDRS website, it allows users to input a PRRSV ORF5 sequence and search for similar sequences in the database. The tool was and continues to be updated in real time providing the capacity of epidemiological trace information, including receive date, geographical location (state), lineage, variant, RFLP, and nucleotide identity, offering a comprehensive view of sequence relationships. Its intuitive interface and streamlined search capabilities made it a valuable resource for researchers, veterinarians, and other stakeholders involved in PRRSV-related studies.

## 4 Discussion

This work has created an organized hub for PRRSV ORF5 sequence data collection and information reporting from participant VDLs. While the underlying data is producer, veterinarian, and VDL clientele anonymized and participant VDL proprietary, the generated information has allowed describing the megatrends of PRRSV ORF5 detection in the US. With a total of 115,643 sequences included in the database, this project is positioned as one of the largest PRRSV ORF5 sequences database in the US and globally. Generated information has also been coupled with a BLAST search tool that allows users to provide a given sequence and perform a real-time epidemiological investigation of where and when similar sequences have been detected. Including labs from diverse regions across the country is imperative to obtain a comprehensive understanding of novel sequence distributions nationwide. Future integration of participant labs from various geographical areas may change the landscape of novel sequence detections, offering insights into regional disparities and facilitating more nuanced analyses of PRRSV epidemiology nationally. The developed SDRS PRRSV sequencing database not only encapsulates past discoveries but also holds the promise of driving future advancements in the understanding and management of PRRSV disease epidemiology. Its expansion and utilization represent vital steps toward combating this significant animal health production disease effectively.

In analyzing the SDRS PRRSV sequencing database, the overall quality of sequences has remained high, as evidenced by 731 incomplete sequences (0.72%) among the 115,643 sequences submitted over the years. This consistency reflects the efforts to focus on reporting high-quality sequence data, ensuring that only reliable sequences are included for further PRRSV analysis. To further minimize sequencing interference, criteria such as sequence length and the number of ambiguities were used to filter the dataset, ensuring that only high-quality sequences were considered in the data analysis. This approach helped reduce the impact of sequencing errors and technical artifacts that could otherwise misrepresent the emergence of sequence trends and novel sequences. The observed decrease in sequences missing swine age groups data over the years (Figure 2) suggests an improvement in metadata recording within VDLs; these improvements have also been reported for capturing RT-PCR data from a subset of the participant VDLs (10). This shift has allowed for more detailed tracking of PRRSV occurrences across various swine age groups, particularly within grow-finish farms, the most significant contributor to the database. The increase in overall sequence submissions may be linked to intensified testing efforts by veterinarians to monitor PRRSV spread and differentiate wild-type from vaccine-like viruses. Understanding how novel PRRSV sequences emerge is complex and influenced by multiple factors, including viral evolution, recombination, and changes in herd management practices. The emergence of novel sequences may result from mutations accumulating over time, reassortment events between different viral strains, or transmission from unrecognized reservoirs. Also, the US swine industry is multi-dynamic, and it is also possible that new strains could have been imported along with swine from other countries like Canada.

Detection of novel sequences revealed a higher number of sequences detected per year in 2010 and 2011, with a noticeable decline in subsequent years. The high number of sequences without state identification, primarily from the early years, reflects the limited metadata recording in the 2000s and early 2010s, with recent improvements seen in these records. The significant reduction in sequences with unknown swine age groups likely reflects improvements in metadata recording systems at VDLs, aligning with trends observed in the PRRSV sequencing database. The elevated numbers of novel sequences detected in the initial 2 years can be attributed to the developmental stage of the PRRSV BLAST database, which at that time lacked a diverse set of PRRSV sequences. The novel sequence identification algorithm relies on previously recorded sequences to determine novel sequences. As more sequences were added over time, the increased diversity in the database enhanced its ability to accurately identify truly novel sequences. Novel sequences were most frequently identified in grow-finish farms (Figure 5), corresponding to the high volume of sequences from this category in the database and emphasizing the critical role of grow-finish farms in PRRSV epidemiology.

The geographical distribution of sequences highlighted Iowa as the primary contributor, a phenomenon largely attributed to its status as the nation’s leading swine-producing state. Followed closely by Minnesota, Missouri, North Carolina, Illinois, Oklahoma, and Indiana, all known for their substantial swine inventory. This prominence may also be supported by the geographical proximity of farms in these states, facilitating frequent interactions such as personal visitors, e.g., maintenance, workers, veterinarians, and animal transfers, which may contribute to spread across sites. Nevertheless, grow-finish sites house the largest swine inventory in the US and have less strict biosecurity practices than breeding herds (45), which may facilitate the spread of PRRSV among sites. The concentration of novel sequence detections in the Midwest emphasized the potential role of dense swine populations and geographic connectivity in identifying novel sequences. Iowa’s prominent share of novel detections is consistent with its dominant presence in the database and status as a swine production hub. Similarly, elevated detections in Minnesota, Illinois, and Indiana likely result from their proximity to Iowa and the frequent movement of animals across state lines. In contrast, states like Tennessee, Kentucky, Ohio, and Pennsylvania reported fewer novel detections. Despite North Carolina’s ranking as the third-largest swine-producing state, its geographic isolation from other major production hubs, outflow of animals for growing purposes, and reduced animal inflow characterized by highly healthy animals for reproduction purposes likely contributes to the lower number of novel sequences identified.

Serum, oral fluid, and lung samples accounted for the majority of specimens with PRRSV ORF5 sequence, marking them as popular choices for testing among veterinarians and researchers (Figure 3). A preference for oral fluid testing in grow-finish farms could be explained by the ease of collection of oral fluids (39, 40) and also by the large usage of oral fluid for RT-PCR testing (10, 39, 40). Historically serum samples were primarily collected from adult/sow farms, but in recent years and after the description of processing fluid samples for PRRSV monitoring and surveillance in breeding herds (41, 42, 46), this sample type has also been used for PRRSV ORF5 sequencing. The fourth-largest category, ‘Unknown’ specimen, likely represents sequences from earlier years when metadata recording practices were less rigorous.

Lineage, variant and RFLP analyses revealed key aspects of PRRSV’s evolutionary dynamics, shedding light into the genetic shifts within the PRRSV population. These analyses were instrumental in tracing the origins and pathways of viral mutations, enabling a better understanding of the virus’s adaptation strategies. Implementing a recently updated lineage classification system (26) reduced the number of unclassified sequences, emphasizing the improved efficiency of the system while also reinforcing the need for continuous updates to address the rapid evolution that PRRSV undergoes. While wild-type sequences dominated, a notable percentage of modified live vaccine-like strains were detected, indicating their usage for promoting the development of immunity for PRRS control and management.

The lineage classification analysis revealed notable trends in both historically prominent and emerging lineages. The surge in detection of L1B sequences among the novel sequences in 2012 and 2013 coincides with the period of heightened activity for this sublineage. Similarly, the sustained activity of L1A since 2015 likely contributed to its prominence among the novel sequences. The resurgence of L1A sequences is aligned with the description of increased production losses and outbreaks associated with this strain (47, 48). L1A (wild-type) has shown consistent declines in detections since 2019. The decrease in the detection of L1A coincides with the rising of sublineage 1C.5 strain (49), which has become the predominant wild-type strain detected in 2024. Sublineage 1H (L1H) exhibited a gradual increase in genetic diversity, potentially accounting for its significant representation. In contrast, other lineages, while present, exhibited low detection rates within the novel sequences, indicating their relatively lower detection or activity during the analyzed period. Novel sequences detected from wild-type lineages, such as L1A and L1H, further reinforce the diversity within wild-type strains and their capacity for genetic adaptation (12, 20, 21, 24, 26). Across the vaccine-like sequences, notable trends were observed over time. The variant 1D.2, belonging to L1D, demonstrated a rise in detections after 2019, coinciding with the introduction of the Prevacent PRRS vaccine that has a PRRSV strain classified as L1D. The variant 5A.1, which belongs to L5A, was the most frequently detected sequence in this sublineage. Its detection likely stems from its association with the Ingelvac MLV vaccine, which has been consistently recovered over the years. Notably, the discontinued commercialization of the MLV Ingelvac ATP is associated with the infrequent detection of this virus in recent years. The detection of 25 novel sequences classified as L5A further highlights the potential continued circulations of wild-type L5A virus or for genetic mutations arising from vaccine strains. These sequences are likely mutations of the vaccine strains, emphasizing the interplay between vaccine use and genetic diversity, however this needs to be tested.

The high number of novel sequences labeled as undetermined variants likely reflects their genetic divergence from established sequences, marking them as outliers within the current classification. Also, the variant classification system has been developed using referent sequences starting from 2019, which may not capture the genetic diversity present in previous years. Similarly, the frequent occurrence of novel sequences assigned a lineage without a specific variant may indicate either strains that faded out before the development of the variant classification system or recent genetic mutations leading to the emergence of new variants within that lineage, particularly as seen with variant 1C-unclassified. This suggests that lineage 1C (L1C) exhibits substantial genetic diversity and variability over time.

The detection of PRRSV-1 has been relatively lower than PRRSV-2. PRRSV-1 occurrences in the US are infrequent and genetically distinct from PRRSV-2. The clinical severity of PRRSV-1 in the US has been mild compared with the PRRSV-2 reducing the interest from veterinarians in further pursuing sequencing of PRRSV-1. Consequently, any mutations detected within PRRSV-1 sequences could meet the criteria for classifying as novel sequences, and among the detected novel sequences, it can be linked to the relatively limited number of PRRSV-1 sequences within the SDRS PRRSV sequencing database. Among PRRSV-1 sequences, an interesting observation is that despite the low number of sublineage L1.1 detection in the SDRS sequencing database, six out of the 16 identified novel PRRSV-1 sequences were classified as L1.1. This pattern may suggest a genetic expansion of this sublineage but without introducing other PRRSV-1 lineages in the US.

The RFLP pattern analysis revealed that most sequences were represented by ten primary patterns, though there has been a shift in the industry from RFLP to lineage-based classification. This trend toward lineage classification is likely positive, as RFLP patterns are sometimes shared across different lineages, potentially creating ambiguity in lineage distinctions and misrepresenting genetic relationships between PRRSV-2 sequences. The RFLP patterns frequently seen among novel sequences correspond to the RFLP patterns most seen in the SDRS sequencing database. The abundance of novel sequences exhibiting RFLP patterns such as 1–4-4, 1–8-4, and 1–7-4 could be attributed to specific lineages known to feature these cut sites prominently. For instance, L1A and L1H are associated with RFLP patterns 1–4-4, 1–8-4, and 1–7-4, which contributed significantly to the detection of novel sequences. The recent emergent sublineage 1C.5 strain is mainly associated with RFLP patterns 1–4-4 and 1–3-4. After a 26-year usage of the RFLP classification system, (23), the assignment of new RFLP patterns has been discontinued. Discontinuing the use of RFLP will likely focus more on lineage or variant classifications; however, continued classification of PRRSV according to contemporary RFLP patterns and its corresponding lineage and variants is necessary for the education of stakeholders and to promote a smooth transition away from RFLP usage.

The notable increase in novel sequence detection and detection frequency during the winter months of December and January was likely due to heightened viral activity in colder temperatures and the seasonal activity of this pathogen, which shows an increased number of positive RT-PCR cases in the winter (10). Interestingly, August showed the highest detection frequency of novel sequences relative to sequences received (Figure 8), possibly due to a smaller volume of testing conducted during summer. Conversely, the lower frequency of novel sequence detections relative to the volume of sequences received in November (Figure 8) could be attributed to the increased activity of predominant PRRSV strains at the same time the PRRSV detection by RT-PCR increased in the US (27, 28).

Evaluating whether novel sequences had the potential to form distinct groups indicated that most novel sequences were singular occurrences, likely reflecting abrupt genetic mutations that were not sustainable over time and, therefore, did not lead to the formation of novel sequence groups. The sequences that formed groups with less than ten detections likely did not persist due to either a limited ability of these sequences to establish in host populations or the presence of more aggressive, competing sequences in the field. Three of the six sequences that formed groups with more than ten detections and some potential for evolution grew into groups with over 20 detections, indicating a capacity for sustained presence. As seen in Table 1, sequences that formed groups had associated variant classification data (1H.28, 1C-unclassified and 1B.4), suggesting they could represent the early stages of new variants within specific lineages (26). These results highlight the capability of SDRS software to identify novel sequences that might otherwise be dismissed as outliers, underscoring its potential in detecting and tracking the emergence of new PRRSV variants. Additionally, the grouping of these sequences with more than 20 detections suggests that their emergence is not merely a result of sequencing artifacts but rather an indication of their active circulation within the swine population. This pattern highlights the realistic rate at which novel sequences are being discovered. An intriguing observation emerged regarding a decline in the detection of novel sequences during specific periods, notably in 2014–15 and 2021. Coinciding with these declines were the ascension of specific sublineages: L1A (RFLP 1–7-4) in 2014–15 and variant 1C.5 (RFLP 1–4-4) in 2020 (49). This observation suggests a compelling link between the emergence of these clinically aggressive strains and the reduced incidence of novel sequence detection. It is probable that these dominant lineages, characterized by heightened virulence, exerted significant pressure of infection during their peak activity, thereby suppressing the emergence of novel variants during those years.

The analysis of novel PRRSV sequences has provided valuable insights into the evolving landscape of this virus within the US swine industry. The observed patterns emphasize the significance of certain farm types, particularly grow-finish farms, and highlight the influence of geographical proximity on the detection of novel sequences. The findings also reveal the crucial role of specific sublineages such as L5A, L1A and L1B, and specific RFLP patterns such as 1–4-4, 1–2-4, 1–7-4 and 1–8-4 in the emergence of novel variants. Notably, those lineages had peaks of detection at different time points and the wild-type predominant strains faded out at some moments. As of December 2024, the L1C.5 represents the majority of wild-type strains detected, and the genetic evolution of this virus could lead to the appearance of new strains that could be either classified according to variants or form clonal expansions that would lead to a future assignment of a new variant name. While the majority of detected sequences appear to be outliers, the identification of sequences with the potential to evolve into more significant strains highlights the importance of continued surveillance and database enhancement. By identifying these trends and providing early warnings of potential risks, the research aims to enhance the industry’s ability to respond to emerging PRRSV threats, thereby safeguarding both animal health and economic stability.

The SDRS PRRSV BLAST tool serves as a valuable resource for both veterinarians and researchers by providing a centralized system for sequence comparison and identification. Veterinarians can utilize the tool to trace the origin date and site state of a novel sequence. The database is updated in real-time and can aid in understanding transmission patterns. By providing a sequence for comparison and receiving back epidemiological information aids decision-makers in implementing targeted disease prevention and control measures. Researchers, on the other hand, can use the tool to identify similar sequences within the SDRS database and detection trends. By offering a single point of access to a broad dataset comparison, the tool improves efficiency and turnaround time by removing the need to contact multiple VDLs individually or create and implement distinct comparison tools within each VDL (50). Similarly, to what happens in other regions, e.g., Denmark,^1^ the SDRS PRRSV BLAST tool streamlines US national access to epidemiological information from PRRSV ORF5 sequences, reducing turnaround time and enhancing efficiency in PRRSV surveillance and animal threat response efforts within U.S. swine production systems.

The SDRS PRRSV sequencing database and associated BLAST database offer support for disease management and prevention efforts. Moreover, beyond its primary function as a data repository, the database boasts crucial metadata linked to each diagnostic sequence data, facilitating multifaceted data analytics. The database comprises both samples for diagnostic as well as surveillance purposes improving its usefulness to identify new sequence emergence. By harnessing these metadata, researchers can delve deeper into various aspects of PRRSV evolution and epidemiology. Additionally, the database can be used for future studies, offering a robust foundation for exploring new avenues in PRRSV research. However, establishing these databases was not without its challenges to ensure that the databases could handle the complexities of PRRSV data, including the need to accommodate a vast and continuously growing volume of sequences. The system was designed to ensure data integrity, consistency, scalability and optimized performance, robust security and access control, comprehensive backup and recovery strategies, continuous performance monitoring, automated testing with continuous integration, and thorough documentation. This ensured that not only was the system scalable and robust but also capable of delivering high performance under varying loads. This attention to detail in both the database structure and software design has resulted in a system that not only supports the current needs of PRRSV surveillance but is also adaptable to future challenges. The significance of the SDRS PRRSV BLAST tool lies in its ability to serve as a centralized platform that consolidates sequences from six major VDLs across the US. This centralization enhances the accessibility and usability of PRRSV sequence data, enabling users to efficiently analyze and interpret sequence similarities across a vast dataset. By offering comprehensive access to this consolidated data, the tool facilitates advanced sequence analysis, supporting ongoing research and disease management efforts in the field of swine health.

Generated epidemiological information could be enhanced by combining the lineage identification with actual production outcomes, e.g., aborts, pre-weaning mortality, nursery or finishing closeout mortality. Identifying and characterizing PRRSV lineage sequences while assessing their direct effects on swine production would be a cutting-edge solution for understanding the implications of emerging strains; however, this capacity was not present in this study. Future studies could explore this aspect to provide a more comprehensive understanding of the relationship between lineage differentiation and production performance. Additionally, the presence of data regarding the exact place and time of origin of the sequences would enhance the epidemiological context. However, identification of the precise location was not possible under this study due to confidentiality restrictions. In outbreak scenarios, a single case can yield numerous PRRSV-2 ORF5 sequences over an extended period due to repeated sampling across different age groups and sample types. Without detailed information linking sequence testing to specific epidemiological events, overrepresentation in certain groupings is possible, which may impact the interpretation of sequence proportions. The availability of site identification incorporating more detailed metadata could enhance the ability to understand disease dynamics and track emerging animal health threats more accurately.

## Data Availability

The datasets used for this study are proprietary of the participant veterinary diagnostic laboratories and its disclosure in raw format is unwarranted by confidentiality agreements. The usage of the data to generate and report macroepedemiological information is provided in this manuscript; the developed SDRS PRRSV BLAST tool, SDRS dashboards, and SDRS monthly PDF reports are all available on the SDRS project website (SDRS, https://www.fieldepi.org/SDRS). Requests to access these datasets should be directed to SDRS, sdrs@iastate.edu.

## Footnotes

^1^ https://prrsv.dk/
